# Pathways to Hypertension Control: Unfinished Journeys of Low‐Income Individuals in Malaysia and the Philippines

**DOI:** 10.1002/hpm.3889

**Published:** 2024-12-28

**Authors:** Benjamin Palafox, Dina Balabanova, Arianna Maever Loreche, Nafiza Mat‐Nasir, Farnaza Ariffin, Mazapuspavina Md‐Yasin, Mohamad‐Rodi Isa, Fadhlina Abd‐Majid, Lia M. Palileo‐Villanueva, Alicia Renedo, Maureen L Seguin, Antonio L Dans, Martin Mckee

**Affiliations:** ^1^ Centre for Global Chronic Conditions London School of Hygiene & Tropical Medicine London UK; ^2^ College of Medicine University of the Philippines Manila Manila Philippines; ^3^ School of Medicine and Public Health Ateneo de Manila University Pasig City Philippines; ^4^ Faculty of Medicine Universiti Teknologi MARA Sungai Buloh Malaysia

**Keywords:** blood pressure, hypertension, low‐income population, Malaysia, non‐communicable disease control, Philippines, primary care

## Abstract

**Background:**

Reducing inequities in hypertension control among those affected in low‐ and middle‐income countries requires person‐centred health system responses based on a contextualised understanding of the choices and care pathways taken by those who rely on the services provided, particularly those from poor and marginalised communities. We examine patterns of care seeking and pathways followed by individuals with hypertension from low‐income households in the Philippines and Malaysia. This study aims to fill a significant gap in the literature by analysing the stages at which individuals make decisions that may affect the successful control of their blood pressure.

**Methods:**

This study presents cross‐sectional survey data collected as part of the Responsive and Equitable Health Systems‐Partnership on Non‐Communicable Diseases (RESPOND) project, a longitudinal observational study in low‐income communities. The study participants were 1191 randomly selected adults aged 35–70 years with a self‐reported history of hypertension or identified as hypertensive through blood pressure screening.

**Results:**

While most low‐income individuals with hypertension in both countries were diagnosed and receiving medication, Malaysians demonstrated higher self‐reported medication adherence. Urban areas in the Philippines showed better hypertension management outcomes compared to rural areas. The study also provides insights into the care seeking pathways followed by low‐income adults diagnosed with hypertension. Nearly half of these individuals in Malaysia and a third in the Philippines were following pathways where they had never changed or stopped treatment without professional advice, and where they were using and adhering to their prescribed medication. Following such pathways was strongly associated with a greater likelihood blood pressure control in the Philippines, but less so in Malayisa.

**Conclusions:**

These findings highlight the need for a contextualised understanding of care seeking choices and the importance of person‐centred solutions. They offer a typology of hypertension care seeking pathways and a foundation for similar research in other settings.

**Summary**

- What is already known on this topic:
  ◦ Effective, equitable and sustainable health system responses to the growing burden of chronic non‐communicable diseases (NCDs) require person‐centred approaches.
  ◦ Tailoring such responses must be informed by a detailed and contextualised understanding of care seeking for NCDs; however, this evidence is often unavailable, especially for those at the margins of society.
- What this study adds:
  ◦ This study illustrates a quantitative method that can characterise the complex ‘therapeutic itineraries’ that individuals with a common NCD follow.
  ◦ Surveys conducted among low‐income individuals with hypertension from Malaysia and the Philippines using this novel approach identified key junctures—that occur both within and outside the health system—of the most common ‘therapeutic itineraries’ followed and provided an understanding of the factors affecting the type of path individuals follow.
- How this study might affect research, practice or policy:
  ◦ The approaches used in this study can help to produce the kinds of evidence required to develop more person‐centred care for those living with NCDs in a wide range of socioeconomic settings.

**Abbreviations**
aRRR: adjusted relative risk ratio
ASEAN: Association of Southeast Asian Nations
cRRR: crude relative risk ratio
CVD: cardiovascular disease
LMIC: low‐ and middle‐income country
MYR: Malaysian Ringgit
NCD: non‐communicable disease
PHP: Philippine Peso
RESPOND: responsive and Equitable Health Systems—Partnership on NCDs
WHO: World Health Organisation
95% CI: 95% confidence interval

## 1 Introduction

Uncontrolled hypertension is an important driver of premature death and disability worldwide, as it is a major risk factor for cardiovascular and other non‐communicable diseases (NCDs) [1]. Although typically considered a ‘disease of the rich’, most of those affected now live in low‐ and middle‐income countries (LMICs) [2], where social safety nets are weak, and the consequences of uncontrolled hypertension can be catastrophic [3, 4].

Yet hypertension is easily diagnosed, with safe and effective treatments available for decades and well‐evidenced guidelines available, including those for low‐resource settings [5, 6]. However, levels of treatment and control are far from ideal in high‐income countries and even worse in LMICs [7, 8], especially among the poor [9]. Inadequately resourced health systems in LMICs struggle to provide access to effective and affordable hypertension care, especially for disadvantaged populations [10]. Systematic reviews have identified many barriers related to the characteristics of patients, providers, and the interaction between them [11], and at the health system level [12]. Improving hypertension control for all is not only a global health priority but also a matter of equitable social and economic progress.

Once diagnosed, hypertension is, for most, a lifelong condition. Diagnosis and treatment initiation are only the first steps on a therapeutic journey. Documents such as the World Heart Federation Hypertension Roadmap describe the optimal pathways, but many people do not follow them for various reasons [13]. A systematic review of factors causing diversions clustered them into three domains: patient‐related (socio‐demographic, knowledge and health beliefs, health status and co‐morbidities, trade‐offs), social (social relationships and traditions) and health system domains (resources and access to hypertension medicines and services) [14]. Given their circumstances, people may follow paths that make sense to them, constructing their own ‘therapeutic itineraries’ that may or may not be optimal [15].

It has been possible to address this unsatisfactory situation using approaches such as packages combining greater use of mid‐level health workers, simplified guidelines, and peer support [16, 17, 18, 19]. However, these strategies should engage with affected communities to co‐design person‐centred solutions [20, 21]. This approach recognises that barriers to care arise from characteristics of both the health system and the patient, so responses should be designed and implemented in a way that fits the lived realities, values and preferences of the people who use them. In doing so, systems must work with patients, their families and communities to foster the trust needed to enable those with chronic conditions, like hypertension, to maintain the long‐term engagement required to manage their condition [15, 22, 23, 24]. This requires in‐depth engagement with those living with chronic conditions, which often reveals how they see their conditions differently from those responsible for policy development and service delivery. For example, a process of co‐creation with people living with diabetes in Peru found that they prioritised measures of the economic impact of their condition and structural support for them over the clinical outcomes used by health professionals [25]. Such processes are part of a growing recognition of the importance of implementation science as a means of closing the ‘know‐do’ gaps that impede the success of strategies to tackle chronic diseases [26].

If the world is to make progress towards Target 3.4 of the Sustainable Development Goals, to ‘reduce by one‐third premature mortality from non‐communicable diseases’ [27] then health system responses must be based on a contextualised understanding of the choices and care pathways taken by those who rely on the services provided, particularly those from poor and marginalised communities, which is currently lacking in the evidence from LMICs [14, 28].

We describe a study in two LMICs, the Philippines and Malaysia, geographically proximal countries that share many characteristics but differ in some important respects (described below). We describe the characteristics, patterns and pathways of care seeking by individuals with hypertension from low‐income households across urban and rural settings, focussing on the steps on the pathway where people change or terminate their treatment. We also examine the determinants of these choices and the consequences for blood pressure control. While it is known that adherence to continuous care is a problem everywhere, here we use a novel approach to follow patients over time to identify their varied trajectories. This offers a template for similar studies elsewhere and fills a gap in the literature.

## 2 Methods

### 2.1 Study Setting

The Responsive and Equitable Health Systems‐Partnership on Non‐Communicable Diseases (RESPOND) project was a mixed‐methods, longitudinal, observational study on treatment seeking for hypertension by adults living in low‐income communities in the Philippines and Malaysia [29].

While both counties have a high prevalence of hypertension [9], we exploited some key differences to elicit the varying effects of context on managing hypertension. Malaysia is an ethnically diverse upper‐middle income country with a well‐developed public sector primary care system funded mainly from general taxation, although with official co‐payments [21]. The Philippines is a populous, archipelagic, lower‐middle income nation with a pluralistic, fragmented health system and relatively high income inequality. The national health insurance programme, PhilHealth, provides limited financial protection primarily for in‐patient care for about 90% of the population via contributions from employers and employees. Health care is delivered in public and private facilities, although the latter are widely used by those who can afford them and are of better quality. Just over 40% of those with hypertension in both countries are on treatment, and 12%–13% are controlled [29]. The authors have reflected on their positions as they analysed and interpreted the findings and a statement on reflexivity is at Appendix 8. A STROBE checklist for the study is at Appendix 9.

### 2.2 Sampling

Briefly, low‐income communities—‘mukim’ in Malaysia and ‘barangays’ in the Philippines with high proportions of households qualifying for government subsidies—were selected in urban and rural strata with probability proportional to size in purposefully selected states (Malaysia) and provinces/cities (Philippines). In Malaysia, these were Selangor, Kelantan, Perak and Johor. In the Philippines, urban communities were in Valenzuela City in Metro Manila and 8 urban and 15 rural communities in Quezon province. Adults living in low‐income households (i.e. qualifying for means‐tested government subsidies under the ‘Bantuan Rakyat 1 Malaysia’ (BR1M) programme in Malaysia and the 4P programme in the Philippines) were randomly selected. *A priori* sample size determination was taken into account by multiple analyses.

Adults aged 35–70 years were eligible if they self‐reported a history of hypertension diagnosis from a health professional, or were identified as hypertensive during blood pressure screening following a standardised procedure based on the World Health Organisation STEPwise approach to Surveillance [30]. Those without a self‐reported history of hypertension were categorised as hypertensive if either the average systolic or diastolic blood pressure was equal to or above 140 mmHg or 90 mmHg, respectively. One among all hypertensive members in eligible households was randomly selected.

### 2.3 Data Collection

On enrolment, trained personnel administered a questionnaire including validated instruments from the Demographic & Health Surveys [31], WHO STEPS [30], World Values Survey [32], and the Living Conditions, Lifestyle and Health Survey [33]. Information collected at individual and household levels included age, gender, marital status, ethnicity, education, occupation, hypertension‐related care experiences (including service utilisation and setting), treatment and adherence, hypertension‐related knowledge and attitudes, tobacco exposure, and other modifiable CVD risk factors. Information on healthcare pathways was collected at discrete stages along the journey (e.g., at the time of diagnosis, changes in treatment, and current practices), as informed by previous research on care pathways [34]. Following piloting, all respondents in the Philippines and a random sub‐sample of respondents in Malaysia also provided information on household income and expenditure, including general health and hypertension management. This analysis uses cross‐sectional baseline data collected in 2018.

### 2.4 Definitions of Core Hypertension Management Indicators

We report four core binary outcomes of hypertension management: (1) awareness/diagnosis, (2) use of current antihypertensive treatment, and (3) blood pressure control among those with hypertension; and (4) self‐reported medication adherence among those on treatment. Current medication use is defined by self‐reports of using any conventional antihypertensive medication in the past 2 weeks, and control as average systolic and diastolic blood pressure less than 140/90 mmHg [35].

Other self‐reported indicators describe the circumstances of hypertension diagnosis; information on follow up, treatment and other advice received at diagnosis; instances and decision‐making on stopping and/or changing hypertension treatment; and measures to monitor and treat hypertension. We created a typology of hypertension care seeking pathways followed by participants, informed by complementary qualitative evidence on ‘therapeutic itineraries’ [15]. Appendix 1 provides definitions of all variables.

### 2.5 Statistical Analysis

We present summary statistics of participant and household characteristics and indicators of hypertension management as percentages and means by country and by urban and rural strata within them. Wald tests assess differences in proportions or means across countries and strata.

We also develop country‐specific (a) multinomial logistic regression models to investigate potential determinants of the type of hypertension care‐seeking pathway followed by our study population; and (b) logistic regression models to assess the potential association between the type of pathway followed and the likelihood of hypertension control. For the multinomial model, the categorical dependent variable identifies which of several pathways the participant followed. Selection of independent variables as potential determinants of pathway selection was informed by existing literature on barriers to hypertension care seeking in LMICs [14, 28]. These include sex, age, highest level of education completed, marital status, employment status, presence of self‐reported co‐morbidities, household size, social capital (proxied by whether or not they knew someone from whom they could borrow cash in a time of need), good knowledge (based on a composite score of 5 questions about the consequences, symptoms and management of hypertension), and self‐reported belief in the effectiveness of both modern and traditional and complementary medicine for the treatment of hypertension. In the logistic model, these covariates are included as potential confounders for the association between the binary dependent variable of hypertension control and the dependent categorical variable for pathway type, as defined above.

Country‐specific models also include community fixed effects to account for clustering arising from unobserved factors that vary by community. These could relate to local health system differences, such as in the availability, affordability, acceptability and quality of hypertension care and medication, or social norms, shared beliefs and practices. As communities were selected within urban and rural areas, an independent variable related to the urban‐rural location was not included in the regression models as it would be perfectly collinear with dummy variables capturing these community‐fixed effects. We present crude and adjusted estimates as relative risk ratios for the multinomial models and odds ratios for the logistic models with their 95% confidence intervals. Each covariate is mutually adjusted for all other potential determinants/confounders described above in multivariable models.

Summary statistics, crude and adjusted relative risk and odds ratios from regression models, and Wald tests are weighted for sampling probability and adjusted for community‐level clustering to account for sampling design. Appendix 2 provides information on derivation of the probability‐based sampling weights.

### 2.6 Patient and Public Involvement

Patients and the public were not directly involved in the design, conduct, reporting, or dissemination plans for this study.

### 2.7 Ethics

Ethical approval was granted by the Observational Research Ethics Committee at the London School of Hygiene & Tropical Medicine (Ref: 12214), and the Research Ethics Boards at the Universiti Putra Malaysia (JKEUPM‐2017‐229), Universiti Teknologi MARA (600‐IRMI(5/1/6) REC/313/18) and the University of the Philippines Manila (UPMREB‐2017‐481‐01). All participants gave informed consent.

## 3 Results

In total 1191 low‐income hypertensive adults were enroled, 606 in the Philippines and 585 in Malaysia. Of these, 889 reported having been diagnosed by a health professional and were included in the analysis, 434 in the Philippines and 455 in Malaysia. We excluded 17 participants from the Philippines and 40 from Malaysia due to missing data. In Malaysia, those excluded did not differ notably from those included in age, education, marital or employment status, time since diagnosis, or household income or size, but included more females and fewer with co‐morbidities. Those excluded in the Philippines did differ from those included on most characteristics, but the small number of excluded participants is unlikely to impact the overall findings of the main analysis. See Appendix 3 in the Supporting Information S1.

### 3.1 Key Hypertension Management Outcomes in the Respond Study Sample

Among all low‐income hypertensive individuals (Table 1), similar proportions in both countries were diagnosed (84% in Malaysia vs. 79% in the Philippines), taking medication (73% vs. 65%) and had their hypertension controlled (22% vs. 24%). However, a higher proportion in Malaysia than in the Philippines self‐reported good medication adherence (99% vs. 66%, respectively; *p* < 0.001).

**TABLE 1:** Key hypertension management outcomes among all low‐income hypertensive adults by country.

Hypertension cascade	Philippines	Malaysia	*p*‐value^a^
Total number hypertensive adults in sample (*N*)	606	585	
% aware of their diagnosis	79.4	83.8	0.118
% currently on medication	65.4	73.2	0.052
% self‐reporting good adherence to their medication^b^	65.6	98.9	<0.001
% with hypertension controlled	22.1	24.4	0.434

^a^ Wald test for a difference in proportions/means (weighted for sampling probability and adjusted for community‐level clustering).

^b^ Among those currently on medication.

Comparing across rural and urban strata, urban areas in the Philippines performed significantly better on each of the key hypertension management indicators than rural areas, apart from adherence (Table 2): 15% points higher for diagnosis, 20% points for treatment, and 10% points for control (all *p* < 0.050). Similar differences were not observed in Malaysia.

**TABLE 2:** Key hypertension management outcomes among all low‐income hypertensive adults by urban and rural area within countries.

Hypertension cascade	Philippines			Malaysia		
	Rural	Urban	*p*‐value^a^	Rural	Urban	*p*‐value^a^
Total number hypertensive adults in sample (*N*)	302	304		293	292	
% aware of their diagnosis	66.1	81.2	0.024	84.0	83.7	0.928
% currently on medication	47.6	67.7	0.011	73.1	73.3	0.966
% self‐reporting good adherence to their medication^b^	76.2	64.6	0.284	98.8	99.1	0.776
% with hypertension controlled	13.5	23.2	0.039	22.4	26.2	0.465

^a^ Wald test for a difference in proportions/means (weighted for sampling probability and adjusted for community‐level clustering).

^b^ Among those currently on medication.

### 3.2 Characteristics of Diagnosed Hypertensive Adults

In both countries, most participants diagnosed with hypertension by the time of data collection were female and married (Table 3). Compared to their Malaysian counterparts, Filipino participants were slightly younger (mean age of 60 years in Malaysia, 56 years in the Philippines), better educated (46% vs. 64% with post‐secondary education), from somewhat larger households (3.8 vs. 4.5 household members), twice as likely to be employed (22% vs. 44%), and less likely to report a co‐morbid condition, such as diabetes (54% vs. 29%). Filipino participants also tended to have been diagnosed with hypertension more recently than those in Malaysia (8.1 mean years since diagnosis in Malaysia vs. 5.8 mean years in the Philippines), and were more likely to report knowing someone from whom they could borrow money in times of need (47% vs. 86%), to believe in the effectiveness of modern medicine (82% vs. 88%) and TCAM (22% vs. 55%) for the treatment of hypertension, but less likely than their Malaysian counterparts to have good knowledge of their condition (57% vs. 40%).

**TABLE 3:** Characteristics of diagnosed hypertensive adults by country.

Characteristic	Philippines	Malaysia	*p*‐value^a^
Sample size of aware hypertensive adults (*N*)	434	455	
% female	73.9	72.6	0.740
Mean age (years)	55.8	59.5	<0.001
% with post‐secondary education	64.2	46.2	<0.001
% married or cohabitating	72.1	72.8	0.791
% currently employed	44.4	22.1	<0.001
Mean number of years since hypertension diagnosis	5.8	8.1	<0.001
% with self‐reported NCD comorbidities	29.3	53.6	<0.001
Median monthly household income (local currency units)^b^	11600	1500	n/a
Mean household size	4.5	3.8	0.030
% self‐reporting knowing someone to borrow money from in time of need	86.0	46.5	0.005
% with good knowledge of hypertension	39.9	56.5	<0.001
% believing in the effectiveness of modern medicine for hypertension treatment	88.3	82.3	0.020
% believing in the effectiveness of traditional and complementary medicine for hypertension treatment	55.2	21.7	<0.001

^a^ Wald test for a difference in proportions/means (weighted for sampling probability and adjusted for community‐level clustering).

^b^ N is lower for this indicator due to refusals and ‘don't know' responses.

Appendix 4 in the Supporting Information S1 provides the same figures by urban‐rural strata in each country. There were no notable differences across strata in Malaysia but more Filipino participants in urban areas were better educated, unemployed, burdened with co‐morbid conditions, and did not believe in the effectiveness of TCAM for the treatment of hypertension.

### 3.3 Care Seeking Patterns of Diagnosed Hypertensive Adults

More detailed information reveals similarities and differences in care seeking (Table 4). While nearly all in both countries were diagnosed with hypertension in a health facility (89% in Malaysia, 98% in the Philippines), just over half in both countries (56% in Malaysia, 62% in the Philippines) went because they were sufficiently worried about their blood pressure. The rest were diagnosed opportunistically during a routine check‐up or other circumstances, such as community hypertension screenings; or incidentally when attending for another condition.

**TABLE 4:** Care seeking patterns of diagnosed hypertensive adults by country.

Cascade step	Detail		Philippines	Malaysia	*p*‐value^a^
Diagnosis	Circumstance of diagnosis	Routine health check‐up	10.3	11.9	0.620
		Visit with a health professional for other reason	11.3	17.9	0.011
		Worried about BP	62.0	56.1	0.257
		Other	16.4	14.1	0.380
	Location of diagnosis	Home	0.5	7.1	0.150
		Health facility	97.9	89.3	0.110
		Retail pharmacy	0.0	0.2	0.262
		Other	1.6	3.4	0.164
	Arranged follow up visit after diagnosis		47.8	98.0	<0.001
		Of whom, attended follow up appointment	69.0	95.2	<0.001
	Treatment prescribed at diagnosis		99.6	95.0	0.021
		Of whom, treatment obtained	98.8	99.9	<0.001
	Lifestyle changes recommended at diagnosis		92.2	92.4	0.889
Treatment	Ever stopped treatment		17.3	16.7	0.818
		Of whom, decision was mine alone	80.3	93.9	0.008
		Of whom, decision was mine in consultation with health professional	3.5	1.0	0.033
		Of whom, decision was made by health professional	16.1	5.0	0.025
	Ever changed treatment		30.3	42.4	0.009
		Of whom, decision was mine alone	7.2	1.1	<0.001
		Of whom, decision was mine in consultation with retail pharmacist	0.0	1.4	0.217
		Of whom, decision was mine in consultation with health professional	5.2	35.5	0.001
		Of whom, decision was mine in consultation with family/friends	1.3	0.0	<0.001
		Of whom, decision was made by health professional	83.7	61.5	0.007
		Of whom, decision was made by TCAM provider	2.7	0.5	0.006
	Currently taking medication		84.2	89.9	0.007
		Of whom, self‐report good adherence	65.5	98.9	<0.001
	Currently making lifestyle changes		86.7	90.8	0.209
Monitoring &	BP measured at least twice annually		95.8	95.1	0.605
Control	Visiting regular provider at least twice annually		55.2	86.1	<0.001
	Type of regular provider^b^	Health professional at hospital	18.2	45.6	0.004
		Health professional at clinic	59.0	49.9	0.201
		Retail pharmacist	0.0	2.0	0.023
		GP at private practice	5.5	2.2	<0.001
		Other private provider	0.8	0.4	0.376
		Community health worker	4.4	0.5	0.009
		TCAM provider	0.1	1.2	0.086
		Family member or friend	19.7	1.6	<0.001
		Any other	4.1	1.1	0.001
	Hypertension controlled		28.1	29.7	0.603

^a^ Wald test for a difference in proportions/means (weighted for sampling probability and adjusted for community‐level clustering).

^b^ Categories of regular provider are not mutually exclusive.

In Malaysia, virtually all attended a follow up appointment after diagnosis, while this was true for only half in the Philippines. Also, at the time of diagnosis, nearly all respondents in both countries were prescribed and obtained their medication and were recommended lifestyle changes.

Regarding treatment, 90% in Malaysia and 84% of diagnosed hypertensives in the Philippines reported currently taking antihypertensive medication. Although only two‐thirds (66%) of those on treatment in the Philippines self‐reported good medication adherence, while nearly all (99%) did so in Malaysia (*p* < 0.001).

Since diagnosis and starting medication, just under a fifth (17%) in both countries reported ever having stopped treatment, mostly of their own accord. More reported ever having changed their treatment (42% in Malaysia, 30% in the Philippines); although, most of these decisions were made either in consultation with or by a health professional.

Most individuals in both countries were also making lifestyle changes (91% in Malaysia, 87% in the Philippines) and having their blood pressure measured at least twice annually (95% in Malaysia, 96% in the Philippines); however, 86% in Malaysia reported visiting a regular health care provider at least twice per year, compared to only 55% in the Philippines (*p* < 0.001). In both countries, most sought regular care from health professionals at hospitals and/or clinics, with only few seeking care from other providers, such as community health workers, retail pharmacists, TCAM practitioners or other private providers, although, 20% in the Philippines reported a family member or friend as a regular provider of care. Yet less than a third of diagnosed low‐income hypertensives in both countries had their blood pressure controlled at data collection (30% in Malaysia, 28% in the Philippines).

Estimates for these care seeking indicators stratified by urban and rural location within countries are in Appendix 5. Briefly, only a few urban‐rural differences were observed. In Malaysia, 33% in rural versus 51% in urban areas reported ever having changed treatment (*p* = 0.001), and in the Philippines, 30% in rural versus 16% in urban areas reported to have ever stopped treatment since being diagnosed (*p* = 0.038).

### 3.4 Pathways of Care Seeking for Hypertension

Based on the evidence above and complementary qualitative evidence from the RESPOND study [15], four characteristics were used to construct care‐seeking pathways for each diagnosed hypertensive adult:

- The circumstance of their diagnosis (i.e., they were diagnosed because they were concerned about their blood pressure vs. they were diagnosed incidentally or opportunistically during a routine check‐up, visit with a health professional for another health concern, or other reason);
- Whether they had ever stopped or changed their treatment since being diagnosed without any advice of a health professional (i.e., the decision was taken alone, in consultation with friends/family or made by a TCAM provider);
- Whether they are currently taking antihypertensive medications, regardless if they had ever stopped or changed treatment since being diagnosed;
- And among those who are on treatment, whether or not they self‐report good adherence to their medication.

**FIGURE 1:**
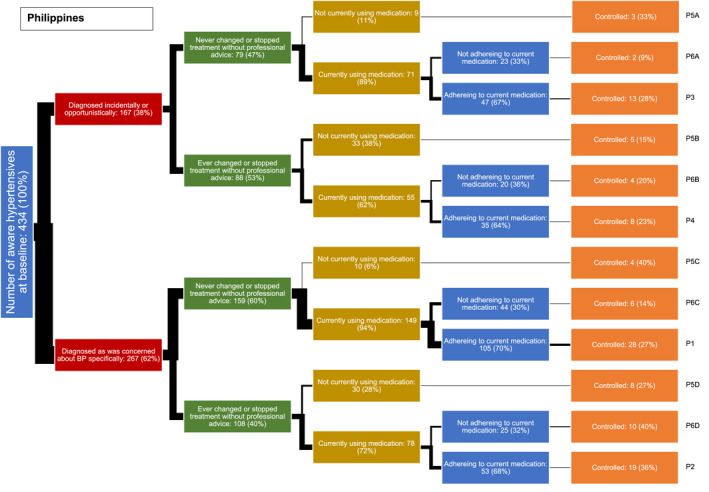
Hypertension care pathways of low‐income adults in the Philippines, *n* (%).

**FIGURE 2:**
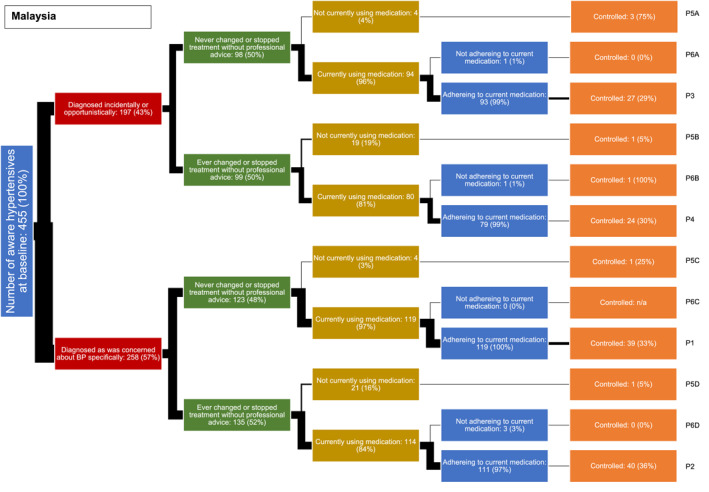
Hypertension care pathways of low‐income adults in Malaysia, *n* (%).

In both countries, we identified a typology of four main hypertension care pathways among low‐income adults (Figures 1 and 2). In each figure the thickness of each line is proportional to the percentage of the sample following it. In both countries, the most common pathway (labelled P1 in the figures) is one where individuals were diagnosed because they were concerned about their blood pressure specifically, had never changed or stopped treatment without professional advice, and who were currently using and adhering to medication (24.9% in the Philippines, 26.9% in Malaysia).

The second most common pathway in both countries (labelled P2) was similar to the first, except that they did report changing or stopping treatment at some point in time without professional advice. In Malaysia, the numbers following this pathway (23.5%) were similar to those following the first pathway type, but only 12.0% followed this pathway in the Philippines.

The third most common pathway type in both countries (labelled P3) is similar to the first, and the fourth pathway type (labelled P4) similar to the second, except on these pathways, individuals were diagnosed incidentally or opportunistically, rather than because they were concerned about their blood pressure; the proportion of people following these third and fourth pathways were similar in both countries (respectively, 11.4% and 6.8% in the Philippines, and 18.7% and 19.8% in Malaysia).

The remaining eight pathways, accounting for a minority of participants in both countries, involved either those not taking medication (four pathways labelled P5A to P5D) or who reported poor adherence (four pathways labelled P6A to P6D).

### 3.5 Determinants of Care Seeking Pathways for Hypertension and the Association With Blood Pressure Control

To examine potential determinants for the type of hypertension care pathway taken by low‐income adults in the Philippines and Malaysia using multinomial logistic regression modelling with community fixed effects, we grouped the pathways described in the previous section into three categories (i.e., dependent or outcome variable):

1. Most desirable pathway types: includes the first and third most common pathways where individuals, once diagnosed, never changed or stopped treatment without professional advice, and are currently using and adhering to medication (P1 and P3 in the figures);
2. Moderately desirable pathway types: includes the second and fourth most common pathways where individuals, once diagnosed, did change or stop treatment without professional advice since diagnosis, but are currently using and adhering to medication (P2 and P4 in the figures); and

Least desirable pathway types: includes the remaining eight pathways, where individuals were either not currently taking medication or reported poor medication adherence if they were taking medication (P5A through P5D and P6A through P6D in the figures).

Using these consolidated pathway types, more low‐income adults diagnosed with hypertension in Malaysia than in the Philippines followed the most desirable (45.5% vs. 36.3%, *p* = 0.043) and moderately desirable (43.3% vs. 18.8%, *p* < 0.001) pathway types; while many more individuals in the Philippines than in Malaysia followed the least desirable pathways (44.9% vs. 11.2%, *p* < 0.001).

The fully adjusted estimates indicate that the pathway type followed by individuals in the Philippines was associated with a greater range of factors than in Malaysia (Table 5). These findings were robust to adjustment for potential confounding (see crude associations in Table 5) and in an alternative specification for the fully adjusted model, which included a covariate for urban‐rural strata, in lieu of the community fixed effects as in our main models (see Appendix 6 for this alternative specification).

**TABLE 5:** Relative risk ratios [and 95% confidence intervals] for the determinants of care seeking pathways for hypertension, by country.

Determinant	Care seeking pathway type	Philippines (*N* = 434)		Malaysia (*N* = 455)	
		Crude	Adjusted	Crude	Adjusted
Female	Least desirable (reference)	1.00	1.00	1.00	1.00
	Moderately desirable	0.82	0.82	1.59	1.40
		[0.59, 1.12]	[0.59, 1.14]	[0.97, 2.61]	[0.90, 2.18]
	Most desirable	1.05	1.11	1.13	1.15
		[0.91, 1.20]	[0.96, 1.27]	[0.86, 1.49]	[0.69, 1.91]
Age 50+ years	Least desirable (reference)	1.00	1.00	1.00	1.00
	Moderately desirable	3.00***	2.43***	2.38	1.95
		[2.15, 4.19]	[1.72, 3.42]	[0.83, 6.85]	[0.33, 11.49]
	Most desirable	2.00***	1.80***	3.25	3.51
		[1.81, 2.21]	[1.62, 2.01]	[0.96, 10.97]	[0.72, 16.96]
Any post‐secondary education	Least desirable (reference)	1.00	1.00	1.00	1.00
	Moderately desirable	1.05	1.07	0.67	0.88
		[0.91, 1.21]	[0.91, 1.24]	[0.30, 1.51]	[0.53, 1.47]
	Most desirable	1.17**	1.20***	0.89	1.20
		[1.04, 1.30]	[1.09, 1.33]	[0.36, 2.22]	[0.57, 2.53]
Married/cohabitating	Least desirable (reference)	1.00	1.00	1.00	1.00
	Moderately desirable	0.41***	0.48***	1.05	1.14
		[0.35, 0.48]	[0.42, 0.55]	[0.33, 3.34]	[0.40, 3.24]
	Most desirable	0.51***	0.53***	1.38	1.39
		[0.47, 0.56]	[0.48, 0.59]	[0.60, 3.13]	[0.49, 3.96]
Currently employed	Least desirable (reference)	1.00	1.00	1.00	1.00
	Moderately desirable	1.00	1.15	0.46*	0.80
		[0.82, 1.22]	[0.95, 1.40]	[0.22, 0.96]	[0.29, 2.22]
	Most desirable	0.96	1.09	0.52**	0.87
		[0.85, 1.09]	[0.98, 1.21]	[0.31, 0.85]	[0.48, 1.57]
Any self‐reported co‐morbid conditions	Least desirable (reference)	1.00	1.00	1.00	1.00
	Moderately desirable	2.03***	1.81***	3.16***	3.03***
		[1.62, 2.54]	[1.59, 2.07]	[1.85, 5.41]	[1.59, 5.79]
	Most desirable	1.90***	1.74***	2.63**	2.72*
		[1.49, 2.42]	[1.38, 2.19]	[1.31, 5.31]	[1.25, 5.95]
Number of household members	Least desirable (reference)	1.00	1.00	1.00	1.00
	Moderately desirable	0.80***	0.86***	1.02	1.00
		[0.78, 0.83]	[0.84, 0.87]	[0.89, 1.18]	[0.88, 1.14]
	Most desirable	0.93***	0.96***	0.95	0.91
		[0.91, 0.94]	[0.95, 0.98]	[0.81, 1.12]	[0.77, 1.08]
Could borrow cash from someone when needed (social capital proxy)	Least desirable (reference)	1.00	1.00	1.00	1.00
	Moderately desirable	1.83***	2.21***	1.63	1.98***
		[1.30, 2.57]	[1.40, 3.51]	[0.84, 3.14]	[1.34, 2.93]
	Most desirable	1.15*	1.33*	1.61	1.54
		[1.02, 1.29]	[1.07, 1.64]	[0.92, 2.81]	[0.92, 2.57]
Good knowledge of hypertension	Least desirable (reference)	1.00	1.00	1.00	1.00
	Moderately desirable	2.08***	2.06***	2.97***	2.56**
		[1.66, 2.59]	[1.61, 2.64]	[1.75, 5.06]	[1.30, 5.02]
	Most desirable	2.22***	2.26***	2.62*	2.00
		[2.15, 2.30]	[2.13, 2.41]	[1.24, 5.53]	[0.91, 4.39]
Believes in the effectiveness of modern medicine for hypertension treatment	Least desirable (reference)	1.00	1.00	1.00	1.00
	Moderately desirable	1.09	1.10	4.59***	5.01***
		[0.87, 1.37]	[0.96, 1.27]	[3.21, 6.56]	[2.81, 8.93]
	Most desirable	1.12*	1.10	4.12***	5.26***
		[1.01, 1.25]	[0.93, 1.30]	[2.89, 5.86]	[3.68, 7.52]
Believes in the effectiveness of TCAM for hypertension treatment	Least desirable (reference)	1.00	1.00	1.00	1.00
	Moderately desirable	0.81**	0.86***	0.20***	0.19***
		[0.69, 0.94]	[0.80, 0.93]	[0.11, 0.36]	[0.11, 0.32]
	Most desirable	1.21***	1.34***	0.18***	0.15***
		[1.08, 1.35]	[1.23, 1.46]	[0.10, 0.32]	[0.06, 0.33]
Constants	Least desirable (reference)		1.00		1.00
	Moderately desirable		0.34***		0.31
			[0.19, 0.60]		[0.03, 3.40]
	Most desirable		0.62***		0.27
			[0.50, 0.77]		[0.03, 2.73]

* for *p* < 0.05, ** for *p* < 0.01, and *** for *p* < 0.001.

In the Philippines, people were more likely to follow any of the more desirable pathway types if they were aged 50 years or older, completed any post‐secondary education, self‐reported any co‐morbid health conditions alongside hypertension, knew someone from whom they could borrow money in times of need, and had good knowledge of hypertension. Conversely, they were comparatively less likely to follow more desirable pathways if they were married, cohabitated with a partner, or lived in larger households. Belief in the effectiveness of TCAM for treating hypertension had a mixed effect: decreasing the likelihood of following a moderately desirable pathway but increasing the likelihood of following one of the most desirable pathways.

In Malaysia, those who self‐reported any co‐morbid health conditions, knew someone from whom they could borrow money in times of need, had good knowledge of hypertension and believed in the effectiveness of modern medicine to treat hypertension were more likely to follow more desirable pathway types, compared to individuals who followed the least desirable pathways. On the other hand, belief in the effectiveness of TCAM for treating hypertension was associated with a decreased likelihood of following any of the more desirable pathways.

Regarding the association between the type of hypertension care seeking pathway followed and the likelihood of blood pressure control, the fully adjusted estimates in the Philippines provide strong evidence that those following any of the more desirable pathways were more likely to achieve blood pressure control, compared to those following one of the least desirable paths (adjusted odds ratios and 95% confidence intervals: 1.42 (1.21, 1.67), *p* < 0.001 for moderately desirable, 1.28 (1.16, 1.41), *p* < 0.001 for most desirable). The associations in Malaysia followed a similar pattern but were not statistically significant at the 5% level (3.07 (0.71, 13.35), *p* = 0.135 for moderately desirable, 2.82 (0.89, 8.92), *p* = 0.078 for most desirable). These associations were also robust in an alternative specification similar to that described above (see Appendix 7 for the full results from the crude, adjusted and alternative model specifications).

While difficult to discern any simple pattern, examining the associations for the community fixed effects that act as a proxy for all local‐level unobserved factors affecting the type of pathway followed indicates that these effects matter. All relative risk ratios corresponding to each study community in the Philippines, and most in Malaysia, were of large magnitude and statistically significant (see Appendix 6 and 7 for complete regression modelling results).

## 4 Discussion

The main contribution of this paper is that it illustrates a quantitative method that can be used to characterise the often complex ‘therapeutic itineraries’ that individuals with a common long‐term condition, such as hypertension, follow [15]. This addresses the understandable tendency in much quantitative research on health seeking behaviour to limit the focus to individual encounters between patients and providers—most commonly diagnosis and initiation of treatment [14]. Our findings reveal that a common feature of the ‘therapeutic itineraries’ followed by low‐income hypertensive individuals in Malaysia and the Philippines occurs outside of the typical patient‐provider interaction, that is the changing or stopping of treatment without consulting a health professional. We have also shown that the type ‘therapeutic itinerary’ followed mattered.

The most desirable pathways represent an ideal therapeutic journey. Individuals on these pathways are either diagnosed due to their proactive concern about their blood pressure or incidentally during routine or opportunistic health checks. Once diagnosed, they consistently adhere to prescribed antihypertensive medication, never stopping or changing treatment without professional guidance. These individuals remain engaged with healthcare providers, maintaining regular follow‐ups and lifestyle changes that promote long‐term control of their condition. This approach demonstrated the strongest correlation with successful blood pressure control, particularly in the Philippines, where continuity of care and adherence are typically weak. This is consistent with some [36], but not all [37] research on the association between continuity of care and health outcomes.

In the moderately desirable pathways, the initial diagnosis and treatment follow a similar trajectory to the most desirable pathways. However, at some point, individuals diverge by stopping or changing their treatment without consulting healthcare professionals. Despite these interruptions, they later resume their prescribed treatment, achieving good adherence. While these pathways are less optimal, they still offer potential for achieving hypertension control, though to a lesser extent than the most desirable paths. They highlight the resilience of some individuals who, despite setbacks, return to treatment and work towards better health outcomes.

The least desirable pathways represent the most challenging journeys. Here, individuals may fail to take medication consistently or adhere poorly to prescribed therapies. This often reflects significant barriers, including economic hardship, health beliefs that conflict with medical advice, or limited access to healthcare resources [38]. These pathways are associated with the lowest rates of blood pressure control, underscoring the critical need to address the systemic and personal challenges that hinder effective management. They illustrate the difficulties faced by marginalised populations in accessing consistent and high‐quality care.

The advent of life‐sustaining treatments for common conditions means that the reality for most patients involves a therapeutic journey they must navigate, with differing degrees of difficulty, to what is, hopefully, the optimal destination. In the case of hypertension, where that destination is defined by long‐term control of their blood pressure, many fall by the wayside. As seen in this study, this is particularly problematic for the poorest groups in society, who often have to balance pressures on time to follow through with treatment and those needed to cope with other daily challenges. The approach taken in this paper offers an analytical model for charting these pathways and understanding how many, and what type of patients follow the different paths. Thus, by developing a typology of pathways and stratifying them according to their conformity with recommended practice, we can start to identify the characteristics of individuals who follow more or less desirable journeys.

In this study we were able to show that in both countries, having good knowledge of hypertension and the presence of comorbidities mattered, which is intuitive given that such individuals are likely to be more informed of their health conditions and how to manage them, and have multiple reasons for engaging with the health system. In both countries, those with higher levels of social capital were also more likely to follow one of the more desirable pathways, which is consistent with other evidence that measures of social capital are associated with narrower inequalities in blood pressure control, but only where health systems are weak, as in many LMIC settings [39]. Our proxy indicator for social capital, knowing someone from whom one could borrow cash when needed, is relevant to our low‐income study population who face many financial barriers to accessing health care. But social capital may take many forms and, therefore, is likely to benefit hypertension care seeking and outcomes in various ways, such as by enhancing knowledge of hypertension and how to manage it, making it easier to access care, and providing informal support that motivates individuals to seek regular care, adhere to medication and maintain long‐term lifestyle changes [15, 22, 39].

In the Philippines, several other factors also mattered for the quality of management. We can speculate that older individuals and those who are better educated may have better access to information, either from official sources or informal networks, or from experience, which may allow them to better navigate the system, and like social capital, helping to compensate for health system weaknesses that are more prevalent in the Philippines than in Malaysia [39]. Those who are married or cohabitating and those with larger families are less likely to follow the most desirable pathway. One possible explanation might be that these individuals have less free time when clinics are operating and, when they do, they may face long queues for facilities with fewer resources [15], but further research is needed to establish if this is the case.

While we must be cautious about over‐interpreting the findings of two relatively small surveys, they do build on what is now extensive evidence on patterns of hypertension diagnosis, treatment and control [7, 40]. As in those studies, we see that hypertension management is suboptimal, and to a greater degree in the Philippines than in Malaysia. Given the challenges of delivering health services in rural areas, with poorer transportation and infrastructure, the urban‐rural gap seen in the Philippines is unsurprising. However, this is not seen in Malaysia, showing that these barriers can be overcome. The basic facts surrounding diagnosis and treatment initiation are broadly similar in both countries, with a few exceptions. However, almost all those given a diagnosis in Malaysia are offered a follow‐up appointment, but this is the case for only half of those in Philippines. Over 85% of patients in Malaysia visit a regular healthcare provider at least twice annually, while this is true for only 55% in the Philippines. In other words, there is much less continuity of care in the Philippines.

Our study has implications for policy and practice. Blood pressure control in the two countries is similar and rather low, given the relatively good access to diagnosis and initiation of treatment. This is surprizing given that in Malaysia there is universal free access to public facilities, extensive screening programmes and affordable medication in the areas studied [21]. In contrast, the Philippine system is more poorly resourced and fragmented. This points to the importance of asking not just whether people have access to care, but what happens when they receive it. Thus, it is clear that even in well‐resourced and generally accessible health systems as in Malaysia, there may be hidden barriers constraining the availability and acceptability of care, particularly affecting follow‐up care and medication adherence [15, 22]. This calls for further examination of beliefs about the nature of hypertension and chronic conditions more generally [23], and other social norms influencing treatment trajectories.

This study highlights the importance of mapping care trajectories and comparing them with what is considered the ideal, seeking to understand what people do at each stage of their journey and where they are most likely to divert or interrupt treatment and why. Eliciting the characteristics of the different types of users following each pathway will make it easier to devise tailored strategies to support individuals and, hopefully, improve hypertension control. Examining the particular constraints faced by the poorest populations, as we do here, is important as they may face different set of problems from the rest of the population, as they are often less trusting of the formal system and take charge of their own pathways [15].

### 4.1 Limitations

Our study has several limitations. Many indicators are derived from self‐reports. Thus, we acknowledge that our estimates are affected by measurement error, as lay understandings of these concepts, particularly of adherence and the chronic nature of hypertension are known to differ from clinical understandings [41, 42]. There may have been misreporting of treatment adherence as participants sometimes substituted conventional medication with traditional and alternative remedies recommended by family and friends [43]. This might contribute to the observation that objectively measured control is low despite respondents reporting that they are taking medication.

Although the surveys are representative of the settings where the study took place, we believe the findings are applicable to other low‐income communities in Malaysia and the Philippines, and, with caution, in other LMICs. Although there were some notable differences in the characteristics of the participants included and excluded in this analysis (Appendix 3 in the Supporting Information S1), the small numbers of those excluded are unlikely to affect our estimates. The median household income, levels of hypertension control, education and employment observed in our samples are closely aligned with national data [7, 44, 45], which suggests that we have, indeed, sampled a suitable cross‐section of hypertensive adults in low‐income communities.

## 5 Recommendations

Our findings point to a series of recommendations that could improve hypertension care for low‐income individuals in Malaysia and the Philippines. Strengthening continuity of care is essential, particularly in the Philippines, where follow‐up rates after diagnosis are low. Facility monitoring systems, such as those used in the WHO HEARTS programme [46] and similar initiatives [47], have been associated with improved blood pressure control. A meta‐analysis of studies in low and middle‐income countries concluded that digital interventions using SMS messaging or smartphone apps can be also effective [48].

Improving access to care, however in ways that is consistent with user circumstances such as the need to work and conduct other essential family functions, is equally vital. Providing subsidised or free antihypertensive medications and expanding healthcare infrastructure in underserved rural areas can address disparities in treatment [49]. This is particularly relevant in the Philippines, where fragmented and poorly resourced systems create significant barriers.

Community health workers and peer supporters can offer patients the support needed to navigate treatment pathways [50].

Cultural beliefs also require attention. Many patients rely on traditional and complementary medicine. This is a contentious area. While policymakers in some countries are seeking ways to integrate traditional practices into formal healthcare, research in Africa has found that those using traditional medicine have worse blood pressure control [51]. Addressing local beliefs about chronic conditions with culturally sensitive interventions and with the help of respected figures in the society may help build trust and improve engagement with mainstream services [24].

Our findings are a reminder that health system responses should adopt person‐centred approaches, tailoring interventions to the unique care‐seeking behaviours in each context, as was done in the successful HOPE‐4 project [19]. Understanding patients' ‘therapeutic itineraries’, as done in this study, can help design solutions that align with their realities [15]. Strengthening primary care services to ensure equitable access and quality, especially in rural and low‐resource settings, is, however, crucial.

Finally, further research and monitoring are needed. Longitudinal studies can track patient outcomes and refine interventions, while implementation research will identify the most effective strategies for scaling solutions. Together, these efforts can create a more responsive, equitable health system that addresses both patient barriers and systemic shortcomings, ultimately improving hypertension control and reducing health inequities.

## 6 Conclusion

Better approaches are needed to describe and understand the complex pathways that individuals follow to manage chronic health conditions, such as hypertension. The novel methods used by this study can help to address this gap by providing the detailed and contextualised evidence required to target limited health systems resources more efficiently, reduce health inequities and contain the growing burden from NCDs in countries at all levels of development.

## Author contributions

B.P. led the analysis and writing of this paper. B.P., D.B., L.M.P.‐V., A.R., M.L.S., A.L.D., K.Y., and M.M. (Principal Investigator) conceived the RESPOND study. All authors contributed to study design, implementation, coordination, interpretation of results, critical review and revision all draughts, and approved the final version of this manuscript. All authors agree to be accountable for all aspects of the work.

## Ethics Statement

Ethical approval for this study was granted by the Observational Research Ethics Committee at the London School of Hygiene & Tropical Medicine (Ref: 12214), the Research Management Centre Universiti Teknologi MARA (600‐IRMI(5/1/6) REC/313/18), the Research Ethics Boards at the Universiti Putra Malaysia (JKEUPM‐2017‐229) and the University of the Philippines Manila (UPMREB‐2017‐481‐01). All methods were performed in accordance with the relevant guidelines and regulations. All participants gave informed consent.

## Consent

The author has nothing to report.

## Conflicts of Interest

L.M.P.‐V. and A.L.D. have been and/or are currently involved in clinical trials of antihypertensive medications that receive some funding from industry. All other author declare that they have no competing interests.

## Supporting information

Supporting Information S1

Supporting Information S2

## Data Availability

The data that support the findings of this study are available on request from the corresponding author. The data are not publicly available due to privacy or ethical restrictions.
