# Supplementary material for: Pathways to Hypertension Control: Unfinished Journeys of Low‐Income Individuals in Malaysia and the Philippines
Source: Int J Health Plann Manage. 2024 Dec 28;40(2):442–57. doi: 10.1002/hpm.3889 (PMC11897856; doi:10.1002/hpm.3889)
Supplement: Supplementary file 1 — Supporting Information S1 [file HPM-40-442-s002.docx]

# SUPPLEMENTAL INFORMATION

## Appendix 1. Definitions of indicators and model covariates

| **Key hypertension management outcomes *among all hypertensives* (Tables 1 and 2)** | |
| --- | --- |
| Hypertensive | Adults aged 35–70 years with self-reported history of hypertension diagnosis from a health professional, or screened to have average systolic or diastolic blood pressure was equal to or above 140 mmHg or 90 mmHg, respectively |
| Aware of diagnosis | Self-reported history of hypertension diagnosis from a health professional among all hypertensives (no vs. yes) |
| Currently on medication | Self-reported to be currently using a specific antihypertensive medication in the preceding 2 weeks among all hypertensives (no vs. yes) |
| Good adherence to medication | Self-reported to be currently adhering to their prescribed antihypertensive medication among those currently using such medication (no vs. yes) |
| Controlled | Those who had an average (across 3 measurements) systolic and diastolic blood pressure of less than 140/90 mmHg among all hypertensives (no vs. yes) |
| **Individual and household characteristics *among aware/diagnosed hypertensives* (Tables 3 and 5)** | |
| Female | Male vs. female sex, self-reported |
| Age | Current age in years, self-reported (categorised as below 50 vs. 50 years or higher) |
| Post-secondary education | Completed only primary, secondary or no education vs. completed any post-secondary education/trade school, self-reported |
| Married/cohabitating | Never married/widowed/separated/divorced vs. currently married/common law/living with partner, self-reported |
| Currently employed | no vs. yes, self-reported |
| Diagnosed with hypertension for at least 5 years | Current number of years since hypertension diagnosis, self-reported (categorised as diagnosed for at least 5 years, no vs. yes) |
| History of NCD comorbidity | Self-reported history of any of the following non-communicable diseases: diabetes mellitus, stroke, heart attack, heart failure, cancer, chronic obstructive pulmonary disease, asthma, valvular heart disease (no vs. yes) |
| Household income | Self-reported from all sources in local currency (Malaysian Ringgit, MYR; Philippine Peso, PHP) |
| Household size | Based on household census |
| Knows someone to borrow money from in time of need | no vs. yes, self-reported |
| Good knowledge of hypertension | Correctly answered at least 4 out of 5 questions about the consequences (can cause stroke or cancer), symptoms (may be asymptomatic) and management of hypertension (medication needed only when blood pressure is high or feel unwell) (no vs. yes) |
| Believes modern medicine is effective to treat hypertension | no vs. yes, self-reported |
| Believes traditional and complementary medicine is effective to treat hypertension | no vs. yes, self-reported |
| Location | Rural vs. urban location, based on national definitions |
| Country | Philippines vs. Malaysia |
| **Care seeking patterns *among aware/diagnosed hypertensives* (Table 4)** | |
| Circumstance of diagnosis | Self-report of the reason or circumstances when the respondent was diagnosed with high blood pressure, including during a routine health check-up, a visit with a health professional for another health condition, because they were specifically worried about their blood pressure, or any other reason |
| Location of diagnosis | Self-report of the location where the respondent was diagnosed with high blood pressure by a health professional, including home, a health facility, a retail pharmacy, or other location |
| Arranged follow up visit after diagnosis | Self-report of whether the respondent was advised to return for a follow up visit at the time of diagnosis (no vs. yes)  Of whom, self-report of whether the respondent attended follow up appointment (no vs. yes) |
| Treatment prescribed at diagnosis | Self-report of whether the respondent was advised to take any modern medications for their high blood pressure at the time of diagnosis (no vs. yes)  Of whom, self-report of whether the respondent obtained or purchased the medication (no vs. yes) |
| Lifestyle changes recommended at diagnosis | Self-report of whether the respondent was advised to make any of the following lifestyle changes for their high blood pressure at the time of diagnosis: quit smoking, reduce alcohol intake, reduce salt (sodium) intake, eat more fruit and vegetables, eat less fatty/fried food, eat/drink fewer sugary beverages/sweets, lose weight, do more exercise or sports, reduce stress (no vs. yes) |
| Ever stopped treatment | Self-report of whether the respondent had ever stopped taking medications altogether since they were first diagnosed with high blood pressure and started taking medications (no vs. yes)  Of whom, self-report of whether the decision was: made by the respondent alone, made by the respondent in consultation with a health professional, made by health professional (no vs. yes) |
| Ever changed treatment | Self-report of whether the respondent’s prescription for high blood pressure medication had ever changed since they were first diagnosed with high blood pressure and started taking medications, including if initially they were not prescribed any medications, but then subsequently were (no vs. yes)  Of whom, self-report of whether the decision was: made by the respondent alone, made by the respondent in consultation with a retail pharmacist, made by the respondent in consultation with a health professional, made by the respondent in consultation with family/friends, made by health professional, made by a TCAM provider (no vs. yes) |
| Currently taking medication | Self-report of whether the respondent was currently taking any modern medications for their high blood pressure (no vs. yes)  Of whom, self-report of good adherence (no vs. yes) |
| Currently making lifestyle changes | Self-report of whether the respondent was currently making any of the following lifestyle changes for their high blood pressure at the time of diagnosis: quit smoking, reduce alcohol intake, reduce salt (sodium) intake, eat more fruit and vegetables, eat less fatty/fried food, eat/drink fewer sugary beverages/sweets, lose weight, do more exercise or sports, reduce stress (no vs. yes) |
| BP measured at least twice annually | Self-reported to currently having their blood pressure measured by any provider type at least 2 times per year (no vs. yes) |
| Visits regular health provider at least 2 times per year for any reason | Self-reported to visit a regular provider of health care at least 2 times per year (no vs. yes) |
| Type of regular provider | Self-reported to have any (up to two) of the following as their main point of contact and most often visited provider of hypertension care: health professional at hospital, health professional at clinic, retail pharmacist, general practitioner at private practice, other private provider, community health worker, TCAM provider, family member or friend, any other. |
| Hypertension controlled | Those who had an average (across 3 measurements) systolic and diastolic blood pressure of less than 140/90 mmHg among diagnosed hypertensives (no vs. yes) |

## Appendix 2. Derivation of the probability-based sampling weights

Sampling weights are needed to analyse the household survey data to avoid biased estimates due to the multi-stage sampling approach that selected communities in each rural and urban strata with probability proportional to their varying population size, an equal number of households within each community, and one household member to participate among all those eligible within the household. Individual-level weights are calculated by taking the inverse product of the:

- Unconditional probability of selecting the community (mukim/barangay) within the state/province (e.g. mukim population/state population at the time of data collection)
- Conditional probability of selecting household within the community (e.g. number of households screened in mukim/total number of households in mukim)
- Conditional probability of selecting participant from all eligible residents within household (i.e. 1/number of eligible adults screened within the household)

## Appendix 3. Characteristics of study participants excluded from analysis

|  | **Philippines** | | | **Malaysia** | | |
| --- | --- | --- | --- | --- | --- | --- |
| **Characteristic** | **Included** | **Excluded** | **p-value^** | **Included** | **Excluded** | **p-value^** |
| Sample size of aware hypertensive adults (N) | 434 | 17 |  | 455 | 40 |  |
| % female | 73.9 | 49.6 | <0.001 | 72.6 | 90.3 | 0.020 |
| Mean age (years) | 55.8 | 49.1 | 0.002 | 59.5 | 57.7 | 0.560 |
| % with post-secondary education | 64.2 | 21.1 | 0.002 | 46.2 | 57.8 | 0.245 |
| % married or cohabitating | 72.1 | 96.0 | 0.005 | 72.8 | 67.6 | 0.446 |
| % currently employed | 44.4 | 52.8 | 0.078 | 22.1 | 15.8 | 0.538 |
| Mean number of years since hypertension diagnosis | 5.8 | 3.1 | 0.001 | 8.1 | 10.2 | 0.239 |
| % with self-reported NCD comorbidities | 29.3 | 3.4 | 0.004 | 53.6 | 36.0 | 0.015 |
| Median monthly household income (local currency units)* | 11600 | 3200 | n/a | 1500 | 1000 | n/a |
| Mean household size | 4.5 | 4.9 | 0.273 | 3.8 | 4.4 | 0.238 |

*^Wald test for a difference in proportions/means (weighted for sampling probability and adjusted for community-level clustering). *N is lower for this indicator due to refusals and 'don't know' responses*

## Appendix 4. Characteristics of diagnosed hypertensive adults by urban and rural strata

|  | **Philippines** | | | **Malaysia** | | |
| --- | --- | --- | --- | --- | --- | --- |
| **Characteristic** | **Rural** | **Urban** | **p-value^** | **Rural** | **Urban** | **p-value^** |
| Sample size of aware hypertensive adults (N) | 194 | 240 |  | 229 | 226 |  |
| % female | 71.4 | 74.1 | 0.628 | 69.5 | 75.4 | 0.477 |
| Mean age (years) | 57.2 | 55.7 | 0.110 | 59.5 | 59.5 | 0.866 |
| % with post-secondary education | 53.8 | 65.2 | 0.012 | 46.7 | 45.8 | 0.899 |
| % married or cohabitating | 66.0 | 72.7 | 0.037 | 77.4 | 68.8 | 0.007 |
| % currently employed | 56.1 | 43.2 | 0.005 | 22.4 | 21.8 | 0.882 |
| Mean number of years since hypertension diagnosis | 5.4 | 5.9 | 0.183 | 7.3 | 8.8 | 0.001 |
| % with self-reported NCD comorbidities | 16.4 | 30.6 | 0.001 | 52.0 | 55.0 | 0.467 |
| Median monthly household income (local currency units)* | 5000 | 12000 | n/a | 1500 | 1500 | n/a |
| Mean household size | 3.9 | 4.5 | 0.012 | 3.8 | 3.9 | 0.858 |
| % self-reporting knowing someone to borrow money from in time of need | 88.6 | 85.7 | 0.334 | 60.9 | 33.6 | 0.058 |
| % with good knowledge of hypertension | 39.4 | 39.9 | 0.909 | 60.9 | 52.6 | 0.057 |
| % believing in the effectiveness of modern medicine for hypertension treatment | 91.4 | 87.9 | 0.348 | 79.7 | 84.7 | 0.170 |
| % believing in the effectiveness of traditional and/or complementary medicine for hypertension treatment | 69.5 | 53.7 | 0.011 | 24.8 | 18.9 | 0.284 |

*^Wald test for a difference in proportions/means (weighted for sampling probability and adjusted for community-level clustering). *N is lower for this indicator due to refusals and 'don't know' responses*

## Appendix 5. Care seeking patterns of diagnosed hypertensive adults by urban and rural strata

|  |  |  | **Philippines** | | | **Malaysia** | | |
| --- | --- | --- | --- | --- | --- | --- | --- | --- |
| **Cascade step** | **Detail** |  | **Rural** | **Urban** | **p-value^** | **Rural** | **Urban** | **p-value^** |
| Diagnosis | Circumstance of diagnosis | routine health check-up | 9.9 | 10.3 | 0.828 | 14.2 | 9.9 | 0.495 |
|  |  | visit with a health professional for other reason | 9.6 | 11.5 | 0.179 | 15.2 | 20.4 | 0.082 |
|  |  | worried about BP | 63.7 | 61.8 | 0.682 | 55.4 | 56.6 | 0.906 |
|  |  | other | 16.8 | 16.4 | 0.932 | 15.3 | 13.1 | 0.705 |
|  | Location of diagnosis | Home | 1.1 | 0.4 | 0.301 | 2.7 | 11.1 | 0.190 |
|  |  | Health facility | 97.4 | 98.0 | 0.653 | 96.0 | 83.3 | 0.054 |
|  |  | Retail pharmacy | 0.0 | 0.0 | n/a | 0.0 | 0.5 | 0.310 |
|  |  | Other | 1.5 | 1.6 | 0.906 | 1.3 | 5.2 | 0.031 |
|  | Arranged follow up visit after diagnosis |  | 53.7 | 47.2 | 0.406 | 99.7 | 96.4 | 0.376 |
|  |  | Of whom, attended follow up appointment | 67.1 | 69.2 | 0.730 | 92.8 | 97.4 | 0.001 |
|  | Treatment prescribed at diagnosis |  | 99.2 | 99.6 | 0.602 | 96.3 | 93.7 | 0.475 |
|  |  | Of whom, treatment obtained | 99.6 | 98.7 | 0.094 | 99.8 | 100.0 | 0.391 |
|  | Lifestyle changes recommended at diagnosis |  | 91.1 | 92.4 | 0.745 | 90.7 | 93.9 | 0.120 |
| Treatment | Ever stopped treatment |  | 30.5 | 15.9 | 0.038 | 13.8 | 19.2 | 0.171 |
|  |  | Of whom, decision was mine alone | 89.7 | 78.5 | 0.075 | 88.4 | 97.5 | 0.058 |
|  |  | Of whom, decision was mine in consultation with health professional | 7.6 | 2.7 | 0.331 | 0.0 | 1.7 | 0.421 |
|  |  | Of whom, decision was made by health professional | 2.7 | 18.8 | 0.001 | 11.6 | 0.8 | 0.008 |
|  | Ever changed treatment |  | 29.1 | 30.4 | 0.808 | 33.3 | 50.5 | 0.001 |
|  |  | Of whom, decision was mine alone | 8.6 | 7.0 | 0.576 | 0.5 | 1.4 | 0.548 |
|  |  | Of whom, decision was mine in consultation with retail pharmacist | 0.0 | 0.0 | n/a | 1.7 | 1.3 | 0.839 |
|  |  | Of whom, decision was mine in consultation with health professional | 29.2 | 2.8 | 0.098 | 36.5 | 34.9 | 0.861 |
|  |  | Of whom, decision was mine in consultation with family/friends | 0.0 | 1.4 | n/a | 0.0 | 0.0 | n/a |
|  |  | Of whom, decision was made by health professional | 62.1 | 85.8 | 0.176 | 60.0 | 62.4 | 0.810 |
|  |  | Of whom, decision was made by TCAM provider | 0.0 | 3.0 | n/a | 1.3 | 0.0 | 0.283 |
|  | Currently taking medication |  | 75.5 | 85.1 | 0.037 | 89.2 | 90.5 | 0.611 |
|  |  | Of whom, self-report good adherence | 76.5 | 64.4 | 0.269 | 98.7 | 99.0 | 0.782 |
|  | Currently making lifestyle changes |  | 91.4 | 86.3 | 0.114 | 94.1 | 88.0 | 0.221 |
| Monitoring & | BP measured at least twice annually |  | 96.3 | 95.8 | 0.797 | 95.6 | 94.6 | 0.724 |
| Control | Visiting regular provider at least twice annually |  | 56.2 | 55.1 | 0.214 | 85.6 | 86.5 | 0.900 |
|  | Type of regular provider | Health professional at hospital | 27.6 | 17.2 | 0.032 | 45.9 | 45.4 | 0.967 |
|  |  | Health professional at clinic | 54.0 | 59.5 | 0.244 | 48.3 | 51.3 | 0.831 |
|  |  | Retail pharmacist | 0.0 | 0.0 | n/a | 1.4 | 2.6 | 0.479 |
|  |  | GP at private practice | 4.3 | 5.6 | 0.454 | 2.1 | 2.3 | 0.817 |
|  |  | Other private provider | 0.5 | 0.8 | 0.608 | 0.9 | 0.0 | 0.226 |
|  |  | Community Health Worker | 13.6 | 3.4 | 0.101 | 0.2 | 0.8 | 0.519 |
|  |  | TCAM provider | 0.8 | 0.0 | 0.345 | 1.9 | 0.5 | 0.141 |
|  |  | Family member or friend | 24.6 | 19.2 | 0.323 | 2.4 | 0.9 | 0.290 |
|  |  | Any other | 2.0 | 4.3 | 0.076 | 2.4 | 0.0 | <0.001 |
|  | Hypertension controlled |  | 20.0 | 28.9 | 0.116 | 27.3 | 31.8 | 0.462 |

*^Wald test for a difference in proportions/means (weighted for sampling probability and adjusted for community-level clustering). *N is lower for this indicator due to refusals and 'don't know' responses*

## Appendix 6. Full modelling results for the determinants of care seeking pathways for hypertension among diagnosed low-income adults by country (main and alternative specification)

| **Determinant** | **Care seeking pathway type** | **Philippines (N=434)** | | | **Malaysia (N=455)** | | |
| --- | --- | --- | --- | --- | --- | --- | --- |
|  |  | **Crude** | **Adjusted (main)** | **Adjusted (alternative)** | **Crude** | **Adjusted (main)** | **Adjusted (alternative)** |
| Female | Least desirable (reference) | 1.00 | 1.00 | 1.00 | 1.00 | 1.00 | 1.00 |
|  | Moderately desirable | 0.82 | 0.82 | 0.81 | 1.59 | 1.40 | 1.31 |
|  |  | [0.59,1.12] | [0.59,1.14] | [0.60,1.09] | [0.97,2.61] | [0.90,2.18] | [0.85,2.03] |
|  | Most desirable | 1.05 | 1.11 | 1.09 | 1.13 | 1.15 | 1.11 |
|  |  | [0.91,1.20] | [0.96,1.27] | [0.98,1.21] | [0.86,1.49] | [0.69,1.91] | [0.69,1.78] |
| Age 50+ years | Least desirable (reference) | 1.00 | 1.00 | 1.00 | 1.00 | 1.00 | 1.00 |
|  | Moderately desirable | 3.00*** | 2.43*** | 2.43*** | 2.38 | 1.95 | 2.18 |
|  |  | [2.15,4.19] | [1.72,3.42] | [1.72,3.42] | [0.83,6.85] | [0.33,11.49] | [0.33,14.21] |
|  | Most desirable | 2.00*** | 1.80*** | 1.79*** | 3.25 | 3.51 | 3.84 |
|  |  | [1.81,2.21] | [1.62,2.01] | [1.58,2.03] | [0.96,10.97] | [0.72,16.96] | [0.77,19.14] |
| Any post-secondary education | Least desirable (reference) | 1.00 | 1.00 | 1.00 | 1.00 | 1.00 | 1.00 |
|  | Moderately desirable | 1.05 | 1.07 | 1.05 | 0.67 | 0.88 | 0.89 |
|  |  | [0.91,1.21] | [0.91,1.24] | [0.88,1.26] | [0.30,1.51] | [0.53,1.47] | [0.52,1.52] |
|  | Most desirable | 1.17** | 1.20*** | 1.19** | 0.89 | 1.20 | 1.23 |
|  |  | [1.04,1.30] | [1.09,1.33] | [1.05,1.35] | [0.36,2.22] | [0.57,2.53] | [0.56,2.70] |
| Married/cohabitating | Least desirable (reference) | 1.00 | 1.00 | 1.00 | 1.00 | 1.00 | 1.00 |
|  | Moderately desirable | 0.41*** | 0.48*** | 0.49*** | 1.05 | 1.14 | 1.04 |
|  |  | [0.35,0.48] | [0.42,0.55] | [0.42,0.56] | [0.33,3.34] | [0.40,3.24] | [0.38,2.83] |
|  | Most desirable | 0.51*** | 0.53*** | 0.53*** | 1.38 | 1.39 | 1.26 |
|  |  | [0.47,0.56] | [0.48,0.59] | [0.48,0.59] | [0.60,3.13] | [0.49,3.96] | [0.46,3.49] |
| Currently employed | Least desirable (reference) | 1.00 | 1.00 | 1.00 | 1.00 | 1.00 | 1.00 |
|  | Moderately desirable | 1.00 | 1.15 | 1.14 | 0.46* | 0.80 | 0.94 |
|  |  | [0.82,1.22] | [0.95,1.40] | [0.92,1.41] | [0.22,0.96] | [0.29,2.22] | [0.32,2.76] |
|  | Most desirable | 0.96 | 1.09 | 1.09 | 0.52** | 0.87 | 0.98 |
|  |  | [0.85,1.09] | [0.98,1.21] | [0.99,1.21] | [0.31,0.85] | [0.48,1.57] | [0.53,1.84] |
| Any self-reported co-morbid conditions | Least desirable (reference) | 1.00 | 1.00 | 1.00 | 1.00 | 1.00 | 1.00 |
|  | Moderately desirable | 2.03*** | 1.81*** | 1.81*** | 3.16*** | 3.03*** | 2.77*** |
|  |  | [1.62,2.54] | [1.59,2.07] | [1.58,2.08] | [1.85,5.41] | [1.59,5.79] | [1.52,5.04] |
|  | Most desirable | 1.90*** | 1.74*** | 1.73*** | 2.63** | 2.72* | 2.48** |
|  |  | [1.49,2.42] | [1.38,2.19] | [1.36,2.20] | [1.31,5.31] | [1.25,5.95] | [1.28,4.83] |
| Number of household members | Least desirable (reference) | 1.00 | 1.00 | 1.00 | 1.00 | 1.00 | 1.00 |
|  | Moderately desirable | 0.80*** | 0.86*** | 0.85*** | 1.02 | 1.00 | 1.02 |
|  |  | [0.78,0.83] | [0.84,0.87] | [0.84,0.87] | [0.89,1.18] | [0.88,1.14] | [0.87,1.18] |
|  | Most desirable | 0.93*** | 0.96*** | 0.96*** | 0.95 | 0.91 | 0.94 |
|  |  | [0.91,0.94] | [0.95,0.98] | [0.95,0.97] | [0.81,1.12] | [0.77,1.08] | [0.77,1.14] |
| Could borrow cash from someone when needed (social capital proxy) | Least desirable (reference) | 1.00 | 1.00 | 1.00 | 1.00 | 1.00 | 1.00 |
|  | Moderately desirable | 1.83*** | 2.21*** | 2.29*** | 1.63 | 1.98*** | 1.93** |
|  |  | [1.30,2.57] | [1.40,3.51] | [1.52,3.45] | [0.84,3.14] | [1.34,2.93] | [1.23,3.01] |
|  | Most desirable | 1.15* | 1.33* | 1.33** | 1.61 | 1.54 | 1.43 |
|  |  | [1.02,1.29] | [1.07,1.64] | [1.09,1.63] | [0.92,2.81] | [0.92,2.57] | [0.84,2.41] |
| Good knowledge of hypertension | Least desirable (reference) | 1.00 | 1.00 | 1.00 | 1.00 | 1.00 | 1.00 |
|  | Moderately desirable | 2.08*** | 2.06*** | 2.07*** | 2.97*** | 2.56** | 2.74** |
|  |  | [1.66,2.59] | [1.61,2.64] | [1.61,2.67] | [1.75,5.06] | [1.30,5.02] | [1.38,5.41] |
|  | Most desirable | 2.22*** | 2.26*** | 2.25*** | 2.62* | 2.00 | 2.14* |
|  |  | [2.15,2.30] | [2.13,2.41] | [2.14,2.37] | [1.24,5.53] | [0.91,4.39] | [1.00,4.60] |
| Believes in the effectiveness of modern medicine for hypertension treatment | Least desirable (reference) | 1.00 | 1.00 | 1.00 | 1.00 | 1.00 | 1.00 |
|  | Moderately desirable | 1.09 | 1.10 | 1.05 | 4.59*** | 5.01*** | 4.61*** |
|  |  | [0.87,1.37] | [0.96,1.27] | [0.87,1.27] | [3.21,6.56] | [2.81,8.93] | [2.51,8.49] |
|  | Most desirable | 1.12* | 1.10 | 1.07 | 4.12*** | 5.26*** | 4.67*** |
|  |  | [1.01,1.25] | [0.93,1.30] | [0.95,1.21] | [2.89,5.86] | [3.68,7.52] | [3.02,7.23] |
| Believes in the effectiveness of TCAM for hypertension treatment | Least desirable (reference) | 1.00 | 1.00 | 1.00 | 1.00 | 1.00 | 1.00 |
|  | Moderately desirable | 0.81** | 0.86*** | 0.87** | 0.20*** | 0.19*** | 0.18*** |
|  |  | [0.69,0.94] | [0.80,0.93] | [0.79,0.97] | [0.11,0.36] | [0.11,0.32] | [0.11,0.29] |
|  | Most desirable | 1.21*** | 1.34*** | 1.35*** | 0.18*** | 0.15*** | 0.14*** |
|  |  | [1.08,1.35] | [1.23,1.46] | [1.24,1.46] | [0.10,0.32] | [0.06,0.33] | [0.06,0.30] |
| Community 1 (reference fixed effect) | Least desirable (reference) |  | 1 |  |  | 1 |  |
|  | Moderately desirable |  | 1 |  |  | 1 |  |
|  | Most desirable |  | 1 |  |  | 1 |  |
| Community 2 | Least desirable (reference) |  | 1 |  |  | 1 |  |
|  | Moderately desirable |  | 0.26*** |  |  | 0.30*** |  |
|  |  |  | [0.25,0.27] |  |  | [0.24,0.38] |  |
|  | Most desirable |  | 0.16*** |  |  | 0.74** |  |
|  |  |  | [0.16,0.17] |  |  | [0.60,0.91] |  |
| Community 3 | Least desirable (reference) |  | 1 |  |  | 1 |  |
|  | Moderately desirable |  | 0.64*** |  |  | 0.81 |  |
|  |  |  | [0.63,0.65] |  |  | [0.58,1.14] |  |
|  | Most desirable |  | 0.40*** |  |  | 1.38 |  |
|  |  |  | [0.39,0.41] |  |  | [1.00,1.91] |  |
| Community 4 | Least desirable (reference) |  | 1 |  |  | 1 |  |
|  | Moderately desirable |  | 0.57*** |  |  | 0.64*** |  |
|  |  |  | [0.49,0.66] |  |  | [0.49,0.82] |  |
|  | Most desirable |  | 0.36*** |  |  | 0.28*** |  |
|  |  |  | [0.33,0.38] |  |  | [0.21,0.39] |  |
| Community 5 | Least desirable (reference) |  | 1 |  |  | 1 |  |
|  | Moderately desirable |  | 3.74*** |  |  | 0.65* |  |
|  |  |  | [3.55,3.95] |  |  | [0.47,0.91] |  |
|  | Most desirable |  | 1.07*** |  |  | 1.40 |  |
|  |  |  | [1.03,1.10] |  |  | [0.96,2.03] |  |
| Community 6 | Least desirable (reference) |  | 1 |  |  | 1 |  |
|  | Moderately desirable |  | 0.66*** |  |  | 0.64** |  |
|  |  |  | [0.61,0.71] |  |  | [0.49,0.84] |  |
|  | Most desirable |  | 0.49*** |  |  | 1.01 |  |
|  |  |  | [0.47,0.51] |  |  | [0.77,1.33] |  |
| Community 7 | Least desirable (reference) |  |  |  |  | 1 |  |
|  | Moderately desirable |  |  |  |  | 1.50** |  |
|  |  |  |  |  |  | [1.12,2.00] |  |
|  | Most desirable |  |  |  |  | 1.19 |  |
|  |  |  |  |  |  | [0.85,1.65] |  |
| Community 8 | Least desirable (reference) |  |  |  |  | 1 |  |
|  | Moderately desirable |  |  |  |  | 1.98*** |  |
|  |  |  |  |  |  | [1.56,2.51] |  |
|  | Most desirable |  |  |  |  | 1.69** |  |
|  |  |  |  |  |  | [1.19,2.40] |  |
| Rural area (reference fixed effect) | Least desirable (reference) |  |  | 1.00 |  |  | 1.00 |
|  | Moderately desirable |  |  | 1.00 |  |  | 1.00 |
|  | Most desirable |  |  | 1.00 |  |  | 1.00 |
| Urban area | Least desirable (reference) |  |  | 1.00 |  |  | 1.00 |
|  | Moderately desirable |  |  | 0.83 |  |  | 1.94 |
|  |  |  |  | [0.43,1.61] |  |  | [0.97,3.89] |
|  | Most desirable |  |  | 1.06 |  |  | 0.78 |
|  |  |  |  | [0.62,1.82] |  |  | [0.32,1.89] |
| Constants | Least desirable (reference) |  | 1.00 | 1.00 |  | 1.00 | 1.00 |
|  | Moderately desirable |  | 0.34*** | 0.28*** |  | 0.31 | 0.20 |
|  |  |  | [0.19,0.60] | [0.14,0.58] |  | [0.03,3.40] | [0.01,2.61] |
|  | Most desirable |  | 0.62*** | 0.30*** |  | 0.27 | 0.34 |
|  |  |  | [0.50,0.77] | [0.18,0.51] |  | [0.03,2.73] | [0.04,3.17] |

** for p<.05, ** for p<.01, and *** for p<.001*

## Appendix 7. Full modelling results for the association between type of care seeking pathways for hypertension and blood pressure control among diagnosed low-income adults by country (main and alternative specification)

| **Covariate** | **Philippines (N=434)** | | | **Malaysia (N=455)** | | |
| --- | --- | --- | --- | --- | --- | --- |
|  | **Crude** | **Adjusted (main)** | **Adjusted (alternative)** | **Crude** | **Adjusted (main)** | **Adjusted (alternative)** |
| Least desirable path (reference) | 1.00 | 1.00 | 1.00 | 1.00 | 1.00 | 1.00 |
| Moderately desirable path | 1.33*** | 1.42*** | 1.39*** | 3.31 | 3.07 | 2.98 |
|  | [1.25,1.42] | [1.21,1.67] | [1.24,1.55] | [0.91,12.05] | [0.71,13.35] | [0.66,13.52] |
| Most desirable path | 1.25*** | 1.28*** | 1.25*** | 2.96* | 2.82 | 2.82 |
|  | [1.20,1.31] | [1.16,1.41] | [1.19,1.31] | [1.02,8.58] | [0.89,8.92] | [0.84,9.49] |
| Female |  | 1.23*** | 1.26*** |  | 0.92 | 1.03 |
|  |  | [1.15,1.31] | [1.13,1.39] |  | [0.59,1.45] | [0.67,1.58] |
| Age 50+ years |  | 1.42*** | 1.44*** |  | 3.93*** | 3.68*** |
|  |  | [1.29,1.58] | [1.27,1.62] |  | [2.74,5.63] | [2.48,5.44] |
| Any post-secondary education |  | 1.27*** | 1.28*** |  | 1.00 | 1.08 |
|  |  | [1.21,1.33] | [1.25,1.32] |  | [0.61,1.63] | [0.69,1.68] |
| Married/cohabitating |  | 1.27*** | 1.27*** |  | 1.21 | 1.18 |
|  |  | [1.15,1.40] | [1.15,1.40] |  | [0.79,1.85] | [0.79,1.78] |
| Currently employed |  | 1.14*** | 1.15*** |  | 1.05 | 1.00 |
|  |  | [1.06,1.24] | [1.07,1.23] |  | [0.61,1.79] | [0.61,1.64] |
| Any self-reported co-morbid conditions |  | 1.09* | 1.10** |  | 0.95 | 0.96 |
|  |  | [1.00,1.19] | [1.02,1.18] |  | [0.59,1.55] | [0.60,1.54] |
| Number of household members |  | 1.09*** | 1.09*** |  | 1.15* | 1.17** |
|  |  | [1.08,1.11] | [1.08,1.11] |  | [1.02,1.29] | [1.04,1.31] |
| Could borrow cash from someone when needed (social capital proxy) |  | 1.79*** | 1.79*** |  | 1.22 | 1.25 |
|  |  | [1.59,2.01] | [1.60,2.00] |  | [0.70,2.14] | [0.78,2.03] |
| Good knowledge of hypertension |  | 0.95 | 0.95 |  | 0.85 | 0.86 |
|  |  | [0.84,1.07] | [0.83,1.09] |  | [0.48,1.52] | [0.47,1.58] |
| Believes in the effectiveness of modern medicine for hypertension treatment |  | 2.12*** | 2.13*** |  | 1.48 | 1.45 |
|  |  | [1.73,2.59] | [1.76,2.57] |  | [0.83,2.65] | [0.85,2.47] |
| Believes in the effectiveness of TCAM for hypertension treatment |  | 1.02 | 1.02 |  | 1.08 | 1.08 |
|  |  | [0.96,1.10] | [0.93,1.11] |  | [0.59,1.98] | [0.59,1.98] |
| Community 1 (reference fixed effect) |  | 1.00 |  |  | 1.00 |  |
| Community 2 |  | 5.62*** |  |  | 0.60*** |  |
|  |  | [5.46,5.78] |  |  | [0.51,0.71] |  |
| Community 3 |  | 1.32*** |  |  | 0.32*** |  |
|  |  | [1.28,1.36] |  |  | [0.27,0.38] |  |
| Community 4 |  | 3.00*** |  |  | 0.47*** |  |
|  |  | [2.90,3.10] |  |  | [0.39,0.57] |  |
| Community 5 |  | 1.81*** |  |  | 0.89 |  |
|  |  | [1.68,1.96] |  |  | [0.71,1.13] |  |
| Community 6 |  | 3.44*** |  |  | 0.52*** |  |
|  |  | [3.37,3.52] |  |  | [0.40,0.67] |  |
| Community 7 |  |  |  |  | 0.96 |  |
|  |  |  |  |  | [0.68,1.35] |  |
| Community 8 |  |  |  |  | 0.65*** |  |
|  |  |  |  |  | [0.60,0.71] |  |
| Urban area |  |  | 1.58 |  |  | 1.20 |
|  |  |  | [0.91,2.74] |  |  | [0.78,1.83] |
| Constants | 0.34*** | 0.01*** | 0.02*** | 0.15** | 0.03*** | 0.01*** |
|  | [0.30,0.38] | [0.01,0.01] | [0.01,0.04] | [0.05,0.49] | [0.01,0.09] | [0.00,0.04] |

** for p<.05, ** for p<.01, and *** for p<.001*

## Appendix 8. Author reflexivity statement

*1. How does this study address local research and policy priorities?*

The broader programme of research of which this study is a core component, was designed produce robust evidence to inform policies for strengthening national health systems to improve the management of hypertension and cardiovascular disease outcomes in the two study countries, which are key local research and policy priorities highlighted in the Philippine National Unified Health Research Agenda 2017-2022 and among the health research priorities of the 11^th^ Malaysia Plan, 2016-2020.

*2. How were local researchers involved in study design?*

The broader programme of research of which this study is a core component was developed in partnership between the University of the Philippines Manila (UPM), UCSI University and Universiti Teknologi MARA in Malaysia, and the London School of Hygiene & Tropical Medicine in the UK. The three programme leads and research team members based in each of the three countries jointly developed the successful funding application and, subsequently, the detailed protocols for each study component, including the one reported here. Study procedures were adapted to capture key data specific to the contexts of Malaysia and the Philippines based on the expertise of research team members in both countries.

*3. How has funding been used to support the local research team?*

Of the original funding for the broader research programme (1.2 million GBP), approximately 55% was disseminated directly to collaborating institutions in Malaysia and the Philippines with a majority of these funds being used for salary support and capacity building of local research team members (particularly for early career researchers) for the duration of the programme, in-country data collection, community engagement and dissemination of the project findings. All funding allocation decisions, both when preparing the original budget and when considering funds reallocation, were considered by leads based at UPM, UCSI and LSHTM. Additionally, researchers from all country teams have drawn repeatedly on open access publication support offered by the UK-based funder of the broader research programme made available via LSHTM as the main contracting institution in the UK.

*4. How are research staff who conducted data collection acknowledged?*

Data collection for this study was conducted both by local research staff employed directly by collaborating institutions in Malaysia and the Philippines, or by professional contracted specifically for data collection All research staff and professional contractors who conducted data collection were invited to contribute to the production of this study manuscript, and those who chose to contribute are included as co-authors; and those who chose not to contribute are recognised in the ‘Acknowledgement’ text of the manuscript.

*5. Do all members of the research partnership have access to study data?*

Research team members based at all collaborating institutions in Malaysia, the Philippines and the UK have access to the study data.

*6. How was data used to develop analytical skills within the partnership?*

The development of the capacities, including skills in data analysis, of early career researchers based at the collaborating institutions in Malaysia, the Philippines and the UK is a core component of the broader programme of research which produced this study. The development of data analysis skills among early career researchers has been supported mainly through the provision of supervised opportunities to lead on specific analyses and publication manuscripts, and funding for short courses on specific data analysis topics related to quantitative and qualitative methods.

*7. How have research partners collaborated in interpreting study data?*

All the data presented in this study were interpreted collaboratively by research team members based in Malaysia, the Philippines and the UK. Insights from this analysis and how they could inform both policy and research were routinely discussed among members of the research team.

*8. How were research partners supported to develop writing skills?*

For this manuscript and all others arising out of the broader programme of research, early career researchers from all collaborating institutions were provided with opportunities to either lead on the preparation of manuscripts as first authors or as co-authors. Support to develop the writing skills of early career researchers was provided through close supervision and guidance of senior researchers from all collaborating institutions, and in some case, through more formal mentoring arrangements between early career researchers and senior researchers.

*9. How will research products be shared to address local needs?*

Findings from the broader programme of research have been routinely shared via policy briefs and dissemination workshops with national and local stakeholders, using established networks and channels maintained by the local partner institutions. This publication and all others arising coming from the broader programme of research, will be open access.

*10. How is the leadership, contribution and ownership of this work by LMIC researchers recognised within the authorship?*

During the inception phase of the broader programme of research of which this study reports one component, all research collaborators from Malaysia, the Philippines and the UK agreed on a set of principles for co-authorship of publications, and for the equitable distribution of opportunities to lead and contribute to publications across LMIC and HIC collaborators, early career and established research team members, and the gender of the lead author(s), including the possibility of joint first authorship. All LMIC research team members who contributed to this study manuscript are included as co-authors (see section 4 for further details). Nearly two-thirds of co-authors are from Malaysia and the Philippines.

*11. How have early career researchers across the partnership been included within the authorship team?*

The lead author and three other co-authors of this manuscript are early career researchers and are based at collaborating institutions in Malaysia, the Philippines and the UK.

*12. How has gender balance been addressed within the authorship?*

The author team includes 9 female and 5 male individuals. As described in question 10 aboveWe sought to mitigate the gender imbalance originating from most co-authors identifying as female by encouraging constant and open communication in routine team meetings and throughout all study processes.

*13. How has the project contributed to training of LMIC researchers?*

Research capacity building was a core component of the broader programme of research, of which this study forms a component. Specifically, the early career research team members based at LMIC collaborating institutions were provided in-work training opportunities to develop skills and gain experience in data collection, analysis and dissemination through peer-reviewed publications, academic conferences and stakeholder engagement. Through this project, several local early career research team members have been able to produce their first first-author peer-reviewed publication.

*14. How has the project contributed to improvements in local infrastructure?*

This project has not directly contributed to infrastructural improvements in Malaysia or the Philippines.

*15. What safeguarding procedures were used to protect local study participants and researchers?*

All study procedures were approved by the institutional review boards at UPM, UCSI, University Teknologi MARA and LSHTM, following principles of the declaration of Helsinki and the Belmont report. All researchers were provided comprehensive training prior to commencing fieldwork, which included a module on risk minimisation. During fieldwork, participants provided written consent to participate in the project.
